# The lived experiences of adolescents with sickle cell disease in Kingston, Jamaica

**DOI:** 10.3402/qhw.v10.28104

**Published:** 2015-09-03

**Authors:** Andrea Brown Forrester, Antoinette Barton-Gooden, Cynthia Pitter, Jascinth L. M. Lindo

**Affiliations:** The UWI School of Nursing, Faculty of Medical Sciences, University of the West Indies, Kingston, Jamaica

**Keywords:** Adolescence, lived experience, psychosocial, sickle cell disease, Jamaica

## Abstract

**Aim:**

To explore the lived experiences of adolescents with sickle cell disease, in Kingston, Jamaica.

**Method:**

A descriptive qualitative design was used for this research. In-depth interviews were conducted with six adolescents with sickle cell disease at a Sickle Cell Unit operated by the University of the West Indies. Interviews were audiotaped, transcribed, and thematically analyzed.

**Results:**

The majority of the adolescents demonstrated a positive self-concept. They reported strong family, school, and peer support which made them feel accepted. All were actively engaged in social activities such as parties, but had challenges participating in sporting activities. Various coping strategies were utilized to address challenges of the disease including praying, watching television, and surfing the Internet.

**Conclusion:**

Sickle cell disease can be very challenging for the adolescent, but with positive self-concept and increased social support, especially from family and peers, these adolescents were able to effectively cope with their condition and live productive lives.

Sickle cell disease (SCD) is one of the most widespread inherited haemolytic disorders affecting persons globally and contributes to significant mortality and morbidity in endemic areas (CDC, 2010; Modell & Darlison, 2008). This genetic disorder is characterized by crescent-shaped red blood cells that block the circulation of blood to tissues, resulting in tissue hypoxia, multi-organ failure, frequent hospitalizations from painful crises, and other complications (Anie, 2005; Newland, 2008). In Jamaica, 1 in 150 newborns have a form of SCD, with 1 in 300 having homozygous disease (HbSS), the most common and severe type of SCD (Hanchard, Hambleton, Harding, & McKenzie, 2005; Serjeant, 2001, 2004). In the United States, 1 in 500 African-American births and 1 in 36,000 Hispanic-America births are estimated to result in patients affected with SCD (NIH, 2008).

In Jamaica, more children with SCD are surviving childhood and transitioning into adolescence and adulthood (King et al., 2007; Lee, Thomas, Cupidore, Serjeant, & Serjeant, 1995). However, even with advances in management of the disease, SCD can cause havoc on affected individuals, including reducing their life expectancy significantly by approximately 30 years from studies conducted in Jamaica and the United States. Currently, the mean survival rates for men and women with SCD are 53 and 58.5 years, respectively, in Jamaica, and 42 and 48 years, respectively, in the United States (Wierenga, Hambleton, & Lewis, 2001; WHO, 2006). Successful adaptation to chronic diseases is not based solely on improved health services, as persons with SCD have many physical and psychological challenges (Anie, 2005; Forgeron et al., 2010). A significant component of health requires good psychological development that is associated with building healthy psychosocial relationships with family and peers during childhood into adolescence. Also, learning positive coping strategies is crucial in chronic disease management as it reduces depression and strengthens family bonding (Cotton et al., 2009; Morgan et al., 2014).

Relationships are often interrupted by frequent and prolonged hospitalizations in SCD, which may affect bonding with family members and creates strained relationships (Forgeron et al., 2010; Grove, Grove, & Michie, 2013; Noll, Kisha, Reiter-Purtill, Gerhardt, & Vannatta, 2010). As sicklers progress into adolescence and adulthood, complications may contribute to disruption of peer relationships, thus eroding their stability and emotional well-being (Knapp, Quinn, Murphy, Brown, & Madden, 2010). This may be devastating for the adolescents physically and psychologically as during this period, they are establishing meaningful interpersonal relationships and discovering new experiences which may be interrupted frequently by painful crises and other complications (Anie, Egunjobi, & Akinyanju, 2010; Knapp et al., 2010). In an attempt to fit in with peers, adolescents may practice risky behaviours such as having unprotected sex, using alcohol, and smoking (Asnani et al., 2014). Furthermore, reproductive issues to reduce these risky behaviours are seldom discussed with adolescents with chronic disease and often they are not included in many healthcare decisions (Knapp et al., 2010). Also, it is possible that these behaviours could be symptoms of the psychological effects of SCD, such as depression, ineffective coping, poorly constructed self-concept, and decreased self-esteem which are high among this group (Anie, 2005; Chapman et al., 2005; Day & Chismark, 2006; Newland, 2008; Sansom-Daly, Peate, Wakefield, Bryant, & Cohn, 2012). These maladies may exacerbate the symptoms of the disease and sometimes contribute to frequent hospitalizations and depression (Lowe & Gibson, 2005).

Suris, Michaud, and Viner (2004) identified an existent reciprocal relationship between chronic diseases and the adolescent's physical development. In many instances, chronic disease results in delayed growth, which may contribute to body dissatisfaction and low self-esteem (Erskine, 2012; Suris et al., 2004). Adolescents with chronic diseases that are characterized by pain are more likely to be alienated and victimized by peers, and this may lead to non-disclosure of disease to others (Forgeron et al., 2010; Suris et al., 2004). Dyson et al. (2010), reported that disclosure did not confer any advantages among teachers and peers, but may contribute to feelings of alienation and stigmatization by teachers, sometimes healthcare workers, and peers (Anderson & Asnani, 2013; Jenerette & Brewer, 2010; Patel & Pathan, 2005). The psychological and physical burden of SCD sometimes affects school attendance which may inadvertently thwart future employment and relationship prospects and may negatively affect mental health thus leading to higher healthcare utilization (Anderson & Asnani, 2013; Anie, 2005; Asnani, Fraser, Lewis, & Reid, 2010; Day & Chrismark, 2006).

Although life expectancy in SCD has improved, there is limited information about the psychosocial needs of adolescents with SCD in Jamaica as most studies focussed on adults (Anderson & Asnani, 2013; Asnani et al., 2010, 2014). Failure to identify the unique challenges of the adolescent group has been reported by Jamaicans living with SCD (Anderson & Asnani, 2013). Furthermore, adults from rural areas had better physical and mental health scores and perceived fewer limitations in their daily living activities as a result of their disease (Asnani, Ali, Reid, Lipps, & Williams-Green, 2008). Whilst Asnani et al. (2008) explored quality of life in adult Jamaican patients with SCD, adolescents face different challenges. Because of their vulnerability, issues such as stigma, delayed menarche, and delayed physical development may affect them negatively, leading to poor self-concept and risky behaviours. This study sought to investigate lived experience of adolescents with SCD in Jamaica as the literature consistently suggests that adolescents with chronic diseases are more likely to experience psychological issues as they traverse this period of development (Bender, 2006; Grove, Grove, & Michie, 2013; Sawyer, Drew, Yeo, & Britto, 2007). Findings from this study can be used to fill the information gap related to the lived experience of adolescents with SCD in Jamaica and provide healthcare workers with an improved perspective that may enhance service provision and social support.

## Theoretical framework

Erick Erickson's theory on the stages of psychosocial development provided the theoretical underpinnings of this study. Adolescents may experience identity versus role confusion which entails the individual's perception of self in relation to others and suggests that peer acceptance is essential during this period. Non-acceptance of peers during adolescence may result in decreased self-esteem and negative thinking (Miller, 1983). According to Erickson, adolescents at this stage seek to explore who they are. Among adolescents, SCD can be psychologically devastating as they cope with symptoms such as delayed menarche in females and painful erections in males, which may be different from their peers (Serjeant, 2001; Serjeant & Serjeant, 2001; Serjeant, Singhal & Hambleton, 2001). Adolescents at this stage want to be identified with their peers and to feel a sense of belonging, and as such both family and peer relationships are vital in their social development (Newland, 2008; Marcia, 1980)

## Study aim

To explore the lived experiences of adolescents aged 18–19 years with SCD, who attend the Sickle Cell Unit in Kingston, Jamaica.

### Study objectives

- Describe the effects of SCD on the self-esteem of affected adolescents.
- Explore the impact that SCD has on the social development of adolescents with the disease.
- Explore the coping mechanisms used by adolescents with SCD.

## Methodology

### Study design

A descriptive qualitative methodology was used to explore the lived experience of 18–19 year-olds with SCD (Sousa, 2014). This approach allowed the clarification of human experiences by allowing adolescents with SCD to shape and create their own understanding of their lived experiences (Polit & Beck, 2010). This method gave an account of the complexities of the phenomenon, is supported by the intuitiveness of the researcher, and is popular in the nursing literature (Finlay, 2009; Giorgi, 2000). Furthermore, qualitative approaches have been successfully used to explore lived experiences of SCD clients with pain (Adegbola et al., 2012). The interviews were conducted by a graduate student who has not worked with sickle cell patients and therefore was free of the biases associated with close contact with these patients as caregiver. However, no systematic attempt at bracketing was undertaken.

### Study site

The Sickle Cell Unit is the only specialized centre for care of persons with SCD in Jamaica and the first in the English-speaking Caribbean. Currently, there are more than 5000 patients recorded in the database, which included approximately 450 adolescents aged 13–19 years who attended this venue. The population at the clinic includes clients from various socio-economic groups, and almost 70% of attendees were residents of urban Kingston and St. Andrew, St. Thomas, St. Catherine, and the Northern parishes. Rural residents were served by monthly outreach clinics in two parishes in the southern and north-western regions of the island. The mandates of the unit were research, patient care, and education.

### Sampling

Adolescents were sampled purposively to intentionally select individuals with insight into the central phenomenon because they were “information rich” (Creswell, 2008). Giorgi (2000) recommends a minimum of three participants for descriptive analysis to identify variations in perspectives. Six adolescents with SCD, aged 18–19 years, were recruited for the study during routine visits to the institution. The 18–19 years age group was chosen to ensure homogeneity of the range of the participants’ experiences. At age 18, adolescents at the Sickle Cell Unit transition to adult care and are no longer seen by paediatricians. Most adolescents are no longer accompanied by their parents or caregiver for health visits except during periods of illness. This is a critical junction in the life of an adolescent as it comes with greater autonomy and responsibility for self-care and treatment adherence. Clients who met the inclusion criteria attended the Sickle Cell Unit within the last year and had been hospitalized for complications of SCD, or had at least one emergency room visit within the last year. Potential participants were excluded if they were less than 18 or older than 19 years, or had a comorbidity.

### Instruments

A semi-structured interview that was guided by the literature review and additional probes were used to elicit rich data, and interviews were audiotaped. Demographic data were collected, and information pertaining to their knowledge and understanding of their illness, sexual development, coping mechanisms, and support system were explored by asking the following open-ended questions: What has been your experience with SCD?; Probe: How does it affect you?; How do you cope with SCD?; Does SCD affect your relationship with peers, teachers, and family?; How does SCD affect your school life, social life, and relationships? (Erskine, 2012). A team of specialized professionals, supervisors, and peers, scrutinized the items and determined the appropriateness of interview questions to meet the study's objective. Following ethical approval, a pilot study was conducted in a similar population, which confirmed the usefulness of the instruments. This process helped to determine the suitability of the instruments.

### Data analysis

The interviews were audiotaped, and data were transcribed *verbatim* by repeatedly listening to the tapes. The typed transcripts were reviewed sequentially by the researcher and supervisors who probed for accuracy and a deeper understanding of the trends and justifications. Themes were identified by the number of repetitions and similarities of concepts within and among the six transcripts reviewed. During the analysis, some themes were merged to prevent repetition and to strengthen the interpretation. Consensus was obtained among the researcher and supervisory team relating the coherence of the arguments and strength of the conclusion (Graneheim & Lundman, 2004; Sousa, 2014). Interpretations were considered in the context of the articulated theoretical framework grounded in Eric Erikson's theory of development through inductive and deductive analyses (Rennie, 2012).

### Ethicality of the study

Ethical approval was granted by the local Ethics Committee, and permission was received from the Director of the Sickle Cell Unit prior to data collection. Data were collected from participants on their routine visits to the Sickle Cell Unit. The prospective participants were identified by the triage nurse as clients who met the age requirement for study. The researcher introduced the study to each adolescent, and they were given an opportunity to ask questions. Participants were assured that refusal to participate in the study would not affect their care in the unit, and written informed consent was obtained following this discussion. Individual face-to-face, semi-structured interviews were conducted in a private room at the facility. Data collection was conducted by the researcher at the Sickle Cell Unit over a 2-week period in April to June 2011.

## Results

Nine clients who met the inclusion criteria were approached, and six were recruited successfully into the study. The findings of this study represent the perceptions of six adolescents 18–19 years who reported their experiences of living with SCD.

### Participants’ demographic characteristics

Participants were equally represented by sex, were between 18 and 19 years old, and resided in Kingston. Three participants were registered at secondary level educational institutions, two at tertiary education institutions, and one participant reported having only completed the 10th grade of high school. All participants were reportedly diagnosed with SCD for more than 10 years. Most had repeated visits to the emergency room for complications of the disease in the last year including two participants who had been hospitalized once and one who had been hospitalized four times.

#### Positive self-concept

The majority of the participants reported a positive self-esteem during the interviews. Three participants emphatically stated that they did not feel different from others and suggested that SCD didn't delineate who they were. Although they felt good about themselves in the presence of SCD, one respondent explained: “I have to tell myself that I can't let sickle cell take control of me, I have to take control of sickle cell disease.” Female participants expressed increased anxiety due to delayed menarche; however, this did not appear to negatively affect how they viewed themselves. Reportedly, participants’ age of menarche ranged between 14 and 17 years old, 3–6 years after their non-SCD peers. One participant was elated and explained: “I didn't really feel anyway different from other peers, even though they started menstruating long before me. I was glad.”

#### Fear of death

Some participants expressed feelings of frustration, sadness, and depression, especially during periods of painful crises. Findings were similar for male and female participants. At times they felt fearful of dying, especially during severe painful crises, hospitalizations, or when a friend or peer died due to complications of SCD. They indicated that they felt they would die from complications of SCD before age 40 years. This was typified by the following quotes:

whenever I go into crisis I feel like it's my last life … sometimes I say why me?

… they say people with sickle cell disease normally die young, so sometimes when I am really sick I feel like I am going to die—That really scares me …

Others were more positive and believed they would live beyond 40 years old.

SCD and school activities

Participants reported being absent from school at least 10 times within the last year as a result of SCD. Absenteeism was reportedly mainly due to painful crises or parents being protective of their child going to school during certain cold or inclement weather conditions. However, one participant indicated, “Sometimes I didn't go to school; not because I was sick, but because my parents prevent me from getting sick; so like if rain falls they don't send me to school.”

Another participant shared: “I missed school a lot, I had to repeat a form because of sickle cell, and it made my grades go down.”

#### Decreased physical activity

All participants revealed they had challenges in participating in physical activities and most were restricted from partaking in these activities at school due to fear by the teachers.

One participant stated:

I have never taken part in sports at school; not because I was sick, but because the teachers were scared.

#### Being normal

With the exception of sporting activities, most participants were socially engaged. Four participants revealed high levels of social involvement such as attending school and parties.

I don't have a problem with sickle cell disease right now. I am involved in everything that everybody without sickle cell is doing, … basically just being normal.

The participants agreed that being hospitalized sometimes prevented them from being involved in social activities. They tried to prevent hospitalization by treating themselves at home whenever there was a problem as a result of SCD, before going to the hospital.

Two of the six participants interviewed expressed a personal decision to refrain from any sexual or intimate relationship, whereas the others were reportedly involved in intimate relationships and were sexually active. One female participant explained:

Sickle cell nuh really affect me being in a relationship, but few times when I have sex the next day me pain up. Sometimes I tell him hurry up and come because I am feeling pain in the joint, leg or the groin.

Translation to English: “Sickle cell disease does not affect me being in a relationship; but a few times when I have sex I feel severe pain on the following day. Sometimes I get my partner to hurry to his orgasm and end the lovemaking because of joint, leg, and groin pain.”

#### Good peer and teacher relationship

All except one participant reported good interpersonal relationships with their peers and teachers who were supportive and helpful. They felt their teachers treated them differently from their peers; as they would not be punished or reprimanded if they did something wrong in class. They also recalled being teased about having SCD in early childhood but not during their adolescent years.

#### Overprotective family

Generally, participants described their family members as being helpful, supportive, and understanding. This allowed them to have a positive attitude towards themselves and to better cope with their condition. Having overprotective family members that restricted them from wearing certain clothing and being involved in physical activities was common among participants.

#### Coping with SCD

Prayer and spiritual activities were major coping strategies for the respondents. They reportedly believed in God and prayed often, especially when there was an exacerbation of their conditions. They participated in spiritual activities such as fasting and going to church for prayer meetings.

One participant explained:

In challenging times I pray, that is the number one thing I do when I am sick. I pray and ask God to help me.

Other coping strategies that were also used were diversional activities. These strategies were applied during periods of exacerbation of their illness as it helped take their minds off their condition and to feel good about themselves. Such activities included watching television or talking with close friends and families. A participant stated:

… I watch TV and surround myself with people who I am comfortable with; cause most of the times when I have a painful crisis not everyone I want to see me in that state.

#### Non-disclosure

Although most participants reportedly felt good about themselves, they did not like to reveal their diagnosis of SCD to other persons, especially teachers. They felt that if they did, they would be treated differently from their peers. All participants reported that disclosure of their sickle cell status was limited to only close relatives and friends. A male participant retorted:

I don't really like talking about it (SCD); I don't like explaining to people about it all the time, they come and ask you, next time they come and ask you again. They get on my nerves sometimes.

Another participant replied:

I feel better not telling my peers and teachers that I have sickle cell …, when I talk about it at times I cry because I have been through a lot …

#### Family and peer support

Family and peer support provided significant comfort for the participants. They all attested that good family support motivated them and helped them cope with SCD. This was done by providing encouragement for them to stay well and to prevent complications. One participant explained convincingly:

My family is very supportive, especially my mother. Over the years she has been there for me and throughout my illness; and she has been there financially, emotionally …

#### Staying healthy

All participants expressed the need to stay healthy to prevent painful crises and complications of SCD. Health maintenance strategies included some social activity restrictions and specific dress codes due to family member's fear of them getting ill. They believed that adhering to medical and dietary regimes prevented deterioration of their condition and prolonged their lives. One participant exclaimed: “I make sure I have all my medication, drink a lot of water, eat right, and ensure that I exercise, keep fit, and get my normal check-ups.”

## Discussion

The study included six adolescents with both sexes being equally represented. Most of the participants received financial support from their parents. Despite the challenges of SCD, most of the participants reported a positive self-concept. This may be attributed to the participant's family support and was previously reported among patients with SCD (Barakat, Patterson et al. 2007). Increased family support, low conflict levels, and positive peer involvement were associated with good psychological adjustment among adolescents with SCD (Cotton et al., 2009; Knapp et al., 2010). Although respondents mainly felt good about themselves, negative feelings were also evident and were expressed as being frustrated, stressed, and saddened especially during periods of painful crises. This was also reported in the literature in which ambivalence is reported in males and females (Anie et al., 2010).

Adolescents with SCD often fear dying at a young age (Anie, 2005; Anie et al., 2010). This fear has been attributed to the adolescent's experience of complications of SCD, near-death experiences, or the death of a friend or peer with SCD. In this study, anxiety related to fear of death was evident for some participants, especially during their most critical periods of illness. It is also possible that a common misconception that persons with sickle cell will die before reaching age 21 might have contributed or heightened the fear of death. It was evident that the support of family and friends played a pivotal role in helping these adolescents think positively about life.

Although the adolescents had reported school absenteeism due to illness (Schwartz, Radcliffe, & Barakat, 2009), they had academic success. It is possible that family, peer, and teacher support aided this process as the literature shows that academic success is positively associated with social class in SCD (Ezenwosu, Emodi, Ikefuna, Chukwu, & Osuorah, 2013). Unfortunately, social class was not accessed in this study, but it might have contributed to the positive academic outcome reported in this sample.

Most participants reported having no restriction to social activities as a result of SCD and saw themselves as being normal teenagers. At this stage of development according to Erikson, forming intimate relationships is an activity that is highly regarded and is pursued (Knapp et al., 2010). Therefore, it was not surprising that they were also involved in intimate sexual relationships which were commensurate with those of their peers. The Jamaica Reproductive Health Survey reported early sexual initiation for both sexes, with girls having first sexual experience before age 15 years and boys earlier (Jamaica Reproductive Health Survey, 2008a, 2008b). Although this topic was not explored in detail, it was previously reported that adolescents with SCD participated in risky sexual practices and should be counselled on the importance of healthy lifestyles (Asnani et al., 2014). The fear of being alienated may contribute to adolescents participating in early sexual activities to gain peer acceptance. Participants often didn't disclose their health status to their partners because they didn't know how long the relationship would last and feared being treated differently as was reported previously by Bhatt, Reid, Lewis, and Asnani (2011).

Although not evident in the current study, findings from the United States suggest that adolescents with SCD experienced social deprivation due to frequent painful crises and hospitalizations and were teased by their peers who thought they were different from others due to their jaundiced eyes (Anie, Egunjobi, & Akinyanju, 2010; Noll et al., 2010). The adolescents in the present study did not feel different from their peers and though they were reportedly teased by their peers in their early years, this was not a feature of their adolescent years. It is possible that the educational services offered at the unit might have contributed to more social acceptance of SCD and reduction in stigma in the over 40 years of research in Jamaica (King, Knight-Madden, & Reid, 2014).

Discriminatory practices have been reported by persons with SCD, and therefore it is not uncommon for them to avoid disclosure as a coping mechanism (Dyson et al., 2010; Suris et al., 2004). The adolescents studied, felt the less they spoke about their condition to individuals, the better it was for them and that talking about their condition perpetually caused them to experience negative emotions. Hence, they were reluctant to disclose their health status to friends and teachers as they did not want to be treated differently by their peers and teachers. Similar findings have been reported among Nigerians with SCD (Anie et al., 2010). According to Erick Erickson, adolescents need to identify with their peers as peer acceptance is important during this developmental stage and this may explain the reluctance to disclose (Dyson et al., 2010). Adolescent males may also internalize their symptoms as a means of coping with SCD (Erskine, 2012); however, this was not evident in the present study, as adolescents disclosed whatever symptoms they experienced to their parents or school nurse.

Frequent hospitalization often prevented the involvement of adolescents with SCD in social activities such as parties (Forgeron et al., 2010); however, this was not evident in this study, as participants applied prevention strategies to advert hospitalization from vaso-occlusive crisis and other complications of SCD. This implies that as adolescents and their families became more knowledgeable about the factors that trigger a sickle cell crisis and ways of preventing complications, they were better able to cope with the disease. Jamaican patients with SCD have placed it in a particular socio-cultural context which allows them to cope (Anderson & Asnani, 2015). Furthermore, by reducing hospitalizations the adolescents had more opportunities to be involved in social activities. Participants disclosed that their families, especially parents, were very overprotective of them. Although overprotective parental behaviour demonstrates love and support for the adolescent and may reduce complications of SCD, it could also negatively affect their development, especially at a time when they are discovering themselves (Erskine, 2012; Suris et al., 2004).

Adolescents utilized various coping strategies to deal with their illness such as spirituality/praying, non-disclosure, and diversional activities. Other coping strategies included reliance on family and friends (Anderson & Asnani, 2013; Anie et al., 2010; Barakat et al., 2007). In the present study, prayer was one of the major strategies utilized by adolescents to cope with illnesses. This finding was evident in previous studies (Anderson & Asnani, 2013; Newland, 2008) in which adolescents with SCD prayed and had faith in a supreme being which they thought would help them during their illnesses. In populations of African descent, spirituality is an acceptable coping strategy that is highly encouraged and acceptable in times of illness (Anie et al., 2010).

In this study, the family, and particularly the mothers of the respondents, played a pivotal role in the coping mechanisms of the majority of adolescents with SCD as reported elsewhere (Barakat, Patterson, et al., 2007; Barlow & Ellard, 2006). They also took the respondents to the doctor and ensured that they adhered to their prescribed medication regimens. Close friends and family members were essential in the motivation and care of adolescents with SCD (Forgeron et al., 2010). It is evident from this study that adolescents with good family support were better able to cope with their conditions. This may be as a result of the type of family structure, and the encouragement and motivation given to adolescents by family and peers.

The better outcome among Jamaican patients with SCD compared to other centres may be due to the nature of the cohort studies which follow the patients from birth and which have a high patient education component including the recognition of acute splenic sequestration by parents of sicklers (Emond et al., 1985; King et al., 2007, 2014).

### Limitations

The study must be considered as exploratory which can be expanded to include more probing and follow-up questions (Polit & Beck, 2010). It consisted of only six individuals from one specialist healthcare facility in Kingston; therefore, the findings are not generalizable. However, the findings are not intended for generalization but to offer a better understanding of the issues which adolescents with SCD encounter. Furthermore, it would have been useful to include persons from rural Jamaica to provide comparative data, as an earlier study reported a higher quality of life quality of life among SCDs who resided in rural areas of Jamaica (Asnani et al., 2008). The operational definition for adolescent is narrow thus may affect their experiences. It is recommended that in future studies, the adolescent age range be expanded.

## Conclusions and implications for nursing practice

The impact of SCD on adolescents in this study included school absenteeism and hospitalization; however, participants reported positive self-esteem which may be due to peer acceptance and family support. Living with a chronic disease is challenging, but social acceptance and utilizing various coping strategies such as prayer and diversional activities have proven to be effective in managing the disease. Although involvement in sporting activities was a major challenge for these adolescents, nurses can assist by educating parents, teachers, and coaches about SCD and the need to encourage physical activities according to their limitation. The benefits of team sports help to strengthen friendship, promote problem solving, and enhance empathy which can positively impact the lives of adolescents. The role of nurses in the management of chronic diseases is paramount in helping families to cope with complications of SCD and instil hope to the adolescents and their families.

## References

[CIT0001] Adegbola M. A, Barnes D. M, Opollo J. G, Herr K, Gray J, McCarthy A. M (2012). Voices of adults living with sickle cell disease pain. Journal of National Black Nurses’ Association: JNBNA.

[CIT0002] Anderson M, Asnani M (2013). ‘You Just Have to Live with It’ coping with sickle cell disease in Jamaica. Qualitative Health Research.

[CIT0003] Anderson M, Asnani M (2015). ‘The white blood cell always eats the red’: How Jamaicans with sickle cell disease understand their illness. Ethnicity & Health.

[CIT0004] Anie K. A (2005). Psychological complications in sickle cell disease. British Journal of Haematology.

[CIT0005] Anie K. A, Egunjobi F. E, Akinyanju O. O (2010). Psychosocial impact of sickle cell disorder: Perspectives from a Nigerian setting. Global Health.

[CIT0006] Asnani M. R, Stone J. H, Blouin M Sickle cell disease. International encyclopedia of rehabilitation.

[CIT0007] Asnani M. R, Ali S. B, Reid M. E, Lipps G, Williams-Green P (2008). Quality of life in patients with sickle cell disease in Jamaica: Rural-urban differences. The International Electronic Journal of Rural and Remote Health.

[CIT0008] Asnani M. R, Bhatt K, Younger N, McFarlane S, Francis D, Gordon-Strachan G (2014). Risky behaviours of Jamaican adolescents with sickle cell disease. Hematology.

[CIT0009] Asnani M. R, Fraser R, Lewis N. A, Reid M (2010). Depression and loneliness in Jamaicans with sickle cell disease. BMC Psychiatry.

[CIT0011] Barakat L. P, Patterson C. A, Weinberger B. S, Simon K, Gonzalez E. R, Dampier C (2007). A prospective study of the role of coping and family functioning in health outcomes for adolescents with sickle cell disease. Journal of Pediatric Hematology/Oncology: Official Journal of the American Society of Pediatric Hematology/Oncology.

[CIT0012] Barakat L. P, Swartz L. A, Simon K, Radcliffe J (2007). Negative thinking as a coping strategy mediator of pain and internalizing symptoms in adolescents with sickle cell disease. Journal of Behavioral Medicine.

[CIT0013] Barlow J. H, Ellard D. R (2006). The psychosocial well-being of children with chronic disease, their parents and siblings: An overview of the research evidence base. Child: Health, Care & Development.

[CIT0014] Bender B. G (2006). Risk taking, depression, adherence and symptom control in adolescents and young adults with asthma. American Journal of Respiratory Critical Care Medicine.

[CIT0015] Bhatt K, Reid M. E, Lewis N. A, Asnani M. R (2011). Knowledge and health beliefs of Jamaican adolescents with sickle cell disease. Pediatric Blood Cancer.

[CIT0062] Chapman D. P, Perry G. S, Strine T. W (2005). The vital link between chronic disease and depressive disorders. Prev Chronic Dis.

[CIT0016] Centers for Disease Control – National Centre for Chronic Disease Prevention and Health Promotion (2010). http://www.cdc.gov/Features/Sicklecell/.

[CIT0017] Cotton S, Grossoehme D, Rosenthal S. L, McGrady M. E, Humency Roberts Y, Hines J (2009). Religious/spiritual coping in adolescents with sickle cell disease: A pilot study. Journal of Pediatric Hematology/Oncology.

[CIT0018] Creswell J (2008). Educational research: Planning, conducting and evaluating quantitative and qualitative research.

[CIT0019] Day S, Chismark E (2006). The cognitive and academic impact of sickle cell disease. Journal of School of Nursing.

[CIT0020] Dyson S. M, Atkin K, Culley L. A, Dyson S. E, Evans H, Rowley D. T (2010). Disclosure and sickle cell disorder: A mixed methods study of young person with sickle cell at school. Social Science & Medicine.

[CIT0021] Emond A. M, Collis R, Darvill D, Higgs D. R, Maude G. H, Serjeant G. R (1985). Acute splenic sequestration in homozygous sickle cell disease: Natural history and management. The Journal of Pediatrics.

[CIT0022] Erskine R (2012). Adolescent boys with sickle cell disease. Clinical Child Psychology and Psychiatry.

[CIT0023] Ezenwosu O. U, Emodi I. J, Ikefuna A. N, Chukwu B. F, Osuorah C. D (2013). Determinants of academic performance in children with sickle cell anaemia. BMC Pediatrics.

[CIT0024] Finlay L (2009). Debating phenomenological research methods. Phenomenology & Practice.

[CIT0025] Forgeron P. A, King S, Stinson J. N, McGrath P. J, MacDonald A. J, Chambers C. T (2010). Social functioning and peer relationships in children and adolescents with chronic pain: A systematic review. Pain Research and Management.

[CIT0026] Giorgi A (2000). Concerning the application of phenomenology to caring research. Scandanavian Journal of Caring Science.

[CIT0028] Graneheim U. H, Lundman B (2004). Qualitative content analysis in nursing research: Concepts, procedures and measures to achieve trustworthiness. Nurse Education Today.

[CIT0029] Grove J, Grove O, Michie C (2013). The real effect of sickle cell disease on children and adolescents. The West London Medical Journal.

[CIT0030] Hanchard N. A, Hambleton I, Harding R. M, McKenzie C (2005). The frequency of the sickle allele in Jamaica has not declined over the last 22 years. British Journal of Haematology.

[CIT0031] Jamaica Reproductive Health Survey (2008a). Jamaica Family Planning Board (NFPB): Statistical Institute of Jamaica (STATIN); Division of Reproductive Health-Centers for Disease Control and Prevention (CDC).

[CIT0032] Jamaica Reproductive Health Survey (2008b). http://www.jnfpb.org/pdf/AdolescentReproductiveHealth/Background_on_Adolescent_Reproductive_Health.pdf.

[CIT0033] Jenerette C. M, Brewer C (2010). Health-related stigma in young adults with sickle cell disease. Journal of the National Medical Association.

[CIT0034] King L, Fraser F, Forbes M, Grindley M, Ali S, Reid M (2007). Newborn sickle-cell disease screening: The Jamaican experience (1995–2006). Journal of Medical Screening.

[CIT0035] King L, Knight-Madden J, Reid M (2014). Newborn screening for sickle cell disease in Jamaica: A review—Past, present and future. The West Indian Medical Journal.

[CIT0036] Knapp C, Quinn G. P, Murphy D, Brown R, Madden V (2010). Adolescents with life-threatening illnesses. Paediatric Palliative Care.

[CIT0037] Lee A, Thomas P, Cupidore L, Serjeant B, Serjeant G (1995). Improved survival in homozygous sickle cell disease: Lessons from a cohort study. British Medical Journal.

[CIT0038] Lowe G. A, Gibson R. C (2005). Depression in adolescents; new developments. West Indian Medical Journal.

[CIT0061] Marcia J. E (1980). Identity in adolescence. Handbook of adolescent psychology.

[CIT0039] Miller P (1983). Theories of developmental psychology.

[CIT0040] Modell B, Darlison M (2008). Global epidemiology of haemoglobinopathy disorders and derived service indicators. Bulletin of the World Health Organization.

[CIT0041] Morgan K. A. D, Scott J, Parshad-Asnani M, Gibson R. C, O'Garo K. N, Lowe G. A (2014). Association among disease severity, religious coping and depression in a cohort of Jamaicans with sickle-cell disease. Mental Health, Religion & Culture.

[CIT0068] National Heart, Lung, and Blood Institute (2008). Sickle cell anemia. Diseases and Conditions Index.

[CIT0042] Newland J. A (2008). Factors influencing independence in adolescents with sickle cell disease. Journal of Child and Adolescent Psychiatric Nursing.

[CIT0043] Noll R. B, Kiska R, Reiter-Purtill J, Gerhardt C. A, Vannatta K (2010). A controlled, longitudinal study of the social functioning of youth with sickle cell disease. Pediatrics.

[CIT0044] Patel A. B, Pathan H. G (2005). Quality of life in children with sickle cell hemoglobinopathy. Indian Journal of Pediatrics.

[CIT0045] Polit D. F, Beck C. T (2010). Essentials of nursing research: Appraising evidence for nursing practice.

[CIT0047] Rennie D. L (2012). Qualitative research as methodical hermeneutics. Psychological Methods.

[CIT0050] Sansom-Daly U. M, Peate M, Wakefield C. E, Bryant R. A, Cohn R. J (2012). A systematic review of psychological interventions for adolescents and young adults living with chronic illness. Health Psychology.

[CIT0051] Sawyer S. M, Drew S, Yeo M. S, Britto M. T (2007). Adolescents with a chronic condition: Challenges living, challenges treating. The Lancet.

[CIT0052] Serjeant G.R (2001). A guide to sickle cell disease.

[CIT0053] Serjeant G. R (2004). The dilemma of defining clinical severity in homozygous sickle cell disease. Current Hematological Report.

[CIT0054] Serjeant G. R, Serjeant B. E (2001). Sickle cell disease.

[CIT0055] Serjeant G. R, Singhal A, Hambleton I. R (2001). Sickle cell disease and age at menarche in Jamaican girls: Observations from a cohort study. Archives of Disease in Childhood.

[CIT0056] Suris J. C, Michaud P. A, Viner R (2004). The adolescent with a chronic condition: Part 1: Developmental issue. Archives of Disease in Childhood.

[CIT0057] Sousa D (2014). Validation in qualitative research: General aspects and specificities of the descriptive phenomenological method. Qualitative Research in Psychology.

[CIT0058] Schwartz L. A, Radcliffe J, Barakat L. P (2009). Associates of school absenteeism with sickle cell disease. Pediatric blood Cancer.

[CIT0059] Wierenga K. J, Hambleton I. R, Lewis N. A (2001). Survival estimates for patients with homozygous sickle-cell disease in Jamaica: A clinic based population study. Lancet.

[CIT0060] World Health Organization (2006). Sickle-cell anaemia-report by the secretariat 1–5.

